# Examining Material Need Insecurity Among LGBTQ+ Older Adults Across Frontier, Rural & Urban South Texas

**DOI:** 10.1093/geroni/igaf122.1730

**Published:** 2025-12-31

**Authors:** Ian Johnson

**Affiliations:** The University of Texas at San Antonio, San Antonio, Texas, United States

## Abstract

Material-need insecurity—unthreatened access to tangible, basic human needs—is a key social determinant of health. LGBTQ+ individuals experience disproportionately higher rates of such insecurity (e.g., food, housing, medication) across their lifespans compared to cisgender, heterosexual age peers. With 30% of older Texans residing in rural areas, understanding the material needs and protective factors of rural LGBTQ+ older adults is crucial. This presentation examines material-need insecurity among LGBTQ+ adults aged 55 and over in South Texas’s 38 counties. Data includes English and Spanish survey responses collected via area agencies on aging and LGBTQ+ community centers, assessing material security across five domains: housing, food, utilities, technology, and prescription medication. Surveys also gathered information on perceived health, diagnosed health conditions, social isolation, and loneliness. Key informant interviews with survey participants and organizational professionals who assisted in survey distribution supplement the survey data. Findings reveal (1) variations in material security across frontier, rural, and urban communities; (2) health conditions as a moderating factor for both insecurity and isolation; (3) the role of social networks in supporting rural aging queer and trans populations; and (4) community-identified solutions to material insecurity, including promotora-model peer-led interventions. The presentation also offers future research ideas using this dataset in conjunction with publicly-available datasets lacking sexual orientation and gender identity measures.

